# Evaluation of Genotoxic Effects of New Molecules with Possible Trypanocidal Activity for Chagas Disease Treatment

**DOI:** 10.1155/2013/287319

**Published:** 2013-11-07

**Authors:** Francisco V. C. Mello, Alcione S. Carvalho, Mônica M. Bastos, Nubia Boechat, Claudia A. F. Aiub, Israel Felzenszwalb

**Affiliations:** ^1^Department of Biophysics and Biometry, Rio de Janeiro State University, Boulevard 28 de Setembro, 87 Fundos, 4**º** Andar, 20551-030 Rio de Janeiro, RJ, Brazil; ^2^Institute of Pharmaceutical Technology, Oswaldo Cruz Institute, Rua Sizenando Nabuco 100, Manguinhos, 21041-250 Rio de Janeiro, RJ, Brazil; ^3^Department of Genetics and Molecular Biology, Federal University of the State of Rio de Janeiro, Rua Frei Caneca, 95 Centro, 20211-040 Rio de Janeiro, RJ, Brazil

## Abstract

Chagas disease is responsible for a large number of human infections and many are also at risk of infection. There is no effective drug for Chagas disease treatment. The Institute of Pharmaceutical Technology at Fiocruz, Brazil, has designed three nitro analogs of the nitroimidazole-thiadiazole, megazol: two triazole analogs PTAL 05-02 and PAMT 09 and a pyrazole analog PTAL 04-09. A set of *Salmonella enterica* serovar Typhimurium strains were used in the bacterial reverse mutation test (Ames test) to determine the mutagenicity and cytotoxicity of megazol and its nitro analogs. Megazol presented positive mutagenic activity at very low concentration, either with or without metabolic activation S9 mix. The mutagenic response of the analogs was detected at higher concentration than the lowest megazol concentration to yield mutagenic activity showing that new advances can be made to develop new analogs. The micronucleus test with rat macrophage cells was used in the genotoxic evaluation. The analogs were capable of inducing micronucleus formation and showed cytotoxic effects. PTAL 04-09 structural modifications might be better suitable for the design of promising new drugs candidate for Chagas' disease treatment.

## 1. Introduction

Chagas disease is a protozoan zoonosis caused by *Trypanosoma cruzi* which has infected and endangers a large number of people, mainly in Latin America [1]. At the moment, there is no truly effective drug on the market for treating Chagas disease. The drugs most widely used nowadays for the acute phase of the disease are nifurtimox (3-methyl-4-[(5-nitrofurfurilidene)-amine]-tiomorfolin-1,1-dioxide), produced by Bayer Health Care, and benznidazole, produced by Roche as Lampit (2-nitro-N-(phenyl-methyl)-1H-imidazole-1-acetamide). Specific chemotherapy using these drugs has a 50–70% chance of curing patients in the acute phase [2]. Prolonged treatment (more than ten years) of patients in the chronic phase using these drugs yields only an 8–30% cure rate [3, 4]. Besides its low efficacy, patients discontinue the treatment due to nonspecific toxicity. Another important factor is that the resistance of the parasite has built up to these drugs [5].

Megazol (2-amino-5-(1-methyl-5-nitro-2-imidazolyl)-1,3,4-thiadiazole), CAS number 19622-55-0, synthesized in 1968 by Berkelhammer and Asato [6] and characterized by Filardi and Brener [7] is an important nitroheterocyclic nitroimidazole containing the 1,3,4-thiadiazole nucleus and has proved to be a promising alternative due to its trypanocidal activity. Comparative studies of megazol with the two compounds mentioned above (nifurtimox and benznidazole) showed an improved activity profile when used in lower doses [8, 9]. The mechanism of action of megazol has been described as being associated with the inhibition of proteins and DNA synthesis in amastigotes of *Trypanosoma cruzi* [10] and also appears to interfere with the parasite's oxygen metabolism [11].

However, megazol is not used clinically, as mutagenic and carcinogenic activity has been reported and related to its nitro group, which is also responsible for its activity against the protozoan [4, 12–14].

Results of structure-mutagenicity relationship and metabolism studies in *Salmonella enterica *serovar Typhimurium provide a basis for the development of suitable nitro analogous. From this perspective, the aim of this work is to present new nitro analogs of megazol with trypanocidal activity designed using rational drug design strategies [15, 16] in order to obtain substances with a similar pharmacodynamic profile but fewer side effects than megazol by removing its genotoxic effect, in accordance with WHO [17] proposals for the control neglected diseases.

## 2. Materials and Methods

### 2.1. Megazol and Its Nitro Analogs

Megazol (2-amino-5-(1-methyl-5-nitro-2-imidazolyl)-1,3,4-thiadiazole) and its nitro analogs PTAL 05-02 (5-(1-methyl-5-nitro-1H-2-imidazolyl)-1H-1,2,4-triazol-3-amine), PAMT 09 (2-amine-N-(1-methyl-4-nitro-1H-imidazole-5-yl)-5-(trifluoromethyl)-1H-1,2,4-triazole), and PTAL 04-09 (1-(1-methyl-4-nitro-1H-imidazole-5-yl)-1H-pirazole) condensed formulas and IC_50_ values are shown in Table 1.

### 2.2. Bacterial Strains

Table 2 presents the *Salmonella enterica *serovar Typhimurium strains, the type of reversion induced, and their genotype. Strains TA97 and TA98 detect frameshift mutation, TA100 and TA1535 detect base substitutions leading to missense mutations, and TA102 detects transitions and/or transversions base substitutions [18, 19]. The *S. enterica* strains TA98, TA97, TA100, TA1535, and TA102 were provided by Dr. B. N. Ames from the University of California, Berkeley, CA, USA. 

### 2.3. *Salmonella *Reverse Mutation Test

Megazol and its nitro analogs PTAL 05-02, PAMT 09 and PTAL 04-09, were tested following the protocol proposed by Maron and Ames [18] with some adaptations [19, 20]. For each experiment, the tester strain was grown overnight in 10 mL of nutrient broth with antibiotics specified for each strain to a density of 2.0 × 10^9^ cells/mL. 100 *μ*L aliquots of each *S. enterica *strain (Table 2) were preincubated (30 min, 37°C, 150 rpm) with 100 *μ*L of megazol (0.05 to 1.0 *μ*g/mL) or its nitro analogs (0.05 to 1.0 *μ*g/mL for PTAL 05-02 and PAMT 09; 1.0 to 50 *μ*g/mL for PTAL 04-09) at different concentrations and 500 *μ*L S9 mix or the same volume of 0.2 M sodium phosphate buffer (pH 7.4). After 30 min, 2 mL top agar (0.7% agar, 0.6% NaCl, 50 *μ*M L-histidine, 50 *μ*M biotin, pH 7.4, 45°C) was added to the test tubes and the final mixtures were poured into Petri dishes with minimal agar (1.5% agar, Vogel-Bonner salts and 2% glucose). These final mixtures were incubated at 37°C for 72 h, and the *his* ^+^ revertant colonies were counted. The mutagenic index (M.I.) was calculated as the mean of each concentration divided by the mean of the negative control. The analysis of variance (one-way ANOVA and Tukey's HSD post hoc analysis, *P* ≤ 0.01) was used for positive results of this assay. The negative control used was dimethyl sulfoxide (DMSO). The positive controls used for this assay in the absence and presence of S9 mix, respectively, were 4-nitroquinoline-1-oxide (4NQO), CAS number: 56-57-5, purity ≥ 97% and 2-aminoanthracene (2AA), CAS number 613-13-8, purity ≥ 96% for TA97 and TA98; sodium azide (SA), CAS number: 26628-22-8, purity ≥ 99.5% and 2AA for TA100 and TA153; and mitomycin C (MitC), CAS number: 50-07-7, purity ≥ 99% for TA102. All mutagens were purchased from Sigma Co. All experiments were performed in triplicate and repeated twice.

### 2.4. Survival Experiments

To determine the cytotoxic effects of megazol and its nitro analogs, the pre-incubation assay mixture in the bacterial reverse mutation test was diluted in 0.9% NaCl (w/v). This suspension contained 2.0 × 10^3^ cells/mL. An aliquot (100 *μ*L) of this suspension was plated on nutrient agar (0.8% Bacto nutrient broth, 0.5% NaCl, and 1.5% agar). The plates were then incubated at 37°C for 24 h and survival fractions were calculated as % survival compared to the negative group. All experiments were performed in triplicate and repeated twice.

### 2.5. S9 Fractions

An S9 fraction, prepared from livers of Sprague-Dawley rats pretreated with a polychlorinated biphenyl mixture (Aroclor 1254), was purchased from Molecular Toxicology Inc. (Moltox, USA). The S9 metabolic activation mixture (S9 mix) used for the bacterial reverse mutation test and survival test was used as previously described by Maron and Ames [18].

### 2.6. Micronucleus Test

The RAW 264.7 macrophage strain was used from a confluence culture. 950 *μ*L Eagle's Minimum Essential Medium (MEM), 1.8 mM Ca^++^, pH 7.6 (Gibco), was supplemented with 1.76 g/L NaHCO_3_, 0.88 g/L pyruvate, 21.6 mg/L aspartic acid, and 16.8 mg/L L-serine, with fetal bovine serum (FBS) (10%), both at 37°C. 50 *μ*L suspension cells (2 × 10^5^ cells/mL) were added to the 24 wells of a microtiter plate containing a coverslip that had been pre-treated with nitric acid 0.1 M for 15 minutes. This suspension was maintained in Eagle's MEM, 1.8 mM Ca^++^, containing FBS (10%), streptomycin (100 mg/L), and penicillin (70 mg/L). The plates were placed in an incubator with an atmosphere of 5% CO_2_ for 24 hours.

For cell treatment, 100 *μ*L of the three concentrations (1.0, 10, and 100 *μ*g/mL) of the nitro analogs of megazol was added (equivalent of 10% of total volume) and the plates were incubated for 24 hours. After the incubation period, the medium was removed and the cells were rinsed with 1.0 mL Eagle's MEM, 1.8 mM Ca^++^. The medium supplemented with FBS (10%) was added (1.0 mL) and the cells were re-incubated for an additional 24 h in an incubator with an atmosphere of 5% CO_2_. The negative control was DMSO. The positive control was N-methyl-N-nitro-N-nitrosoguanidine (MNNG) at a concentration of 0.5 mM.

The Eagle's MEM, 1.8 mM Ca^++^, was replaced with a cold fixative solution methanol-glacial acetic acid (3 : 1) for 15 min. The fixed cells were rinsed with McIlvaine's buffer (MI buffer: 21.01 g/L citric acid and 35.60 g/L Na_2_HPO_4_, pH 7.5) for 2 min and dried at room temperature. The fixed cells were stained with 4′-6-diamidino-2-phenylindole (DAPI) (0.2 *μ*g/mL) dissolved in MI buffer for 40 min. Cells were washed with MI buffer for 2 min followed by distilled water and dried again at room temperature. To determine the mitotic index, the number of cells with micronuclei and the percentage of necrosis and apoptosis (1000 cells per concentration, quintuplicate) were analyzed in a fluorescence microscope (Reichert Univar) with an excitation wavelength of 350 nm [21, 22]. The analysis of variance (one-way ANOVA and Tukey's HSD post hoc analysis, *P* ≤ 0.01) was used for positive results of this assay. All experiments were performed in quintuplicate and repeated twice.

## 3. Results

### 3.1. Reversion of *Salmonella *Strains in the Presence of Megazol

Table 3 shows the mutagenicity and cytotoxicity induction of megazol in the* Salmonella *reverse mutation test.

Megazol exhibited dose-dependent mutagenic activity for a wide range of concentrations and strain in the absence and presence of S9 mix.

TA97 and TA100 were the most responsive strains in the presence and absence of S9 mix. TA100 was the most responsive strains in terms of mutagenic index. 

However, there was no mutagenic activity in the absence of S9 mix for the strain TA102. Unlike the others strains, TA102 has its excision repair mechanism intact and this might explain the absence of mutagenic activity. On the other hand, there was mutagenic activity in the presence of S9 mix, suggesting the generation of metabolites by S9 mix metabolization. 

### 3.2. Reversion of *Salmonella *Strains in the Presence of Megazol Nitro Analogs

Tables 4, 5, and 6 show the mutagenicity and cytotoxicity induction of the megazol nitro analogs in the *Salmonella *reverse mutation test. The experiments with the nitro analogs were conducted using a single strain in conjunction with each megazol analog and therefore they share the same negative and positive control response.

The nitro analog PTAL 05-02 did not present a positive response for the *Salmonella enterica *serovar Typhimurium strains in the absence or presence of S9 mix, though it did present a cytotoxic response for the bacterial strains TA100 and TA1535, with S9 mix (Table 4).

The nitro analog PAMT 09 did not present a mutagenic response for the *S*.* enterica *strains, but it did present a cytotoxic response for the bacterial strain, with S9 mix (Table 5).

The mutagenicity assays with PTAL 04-09 presented a positive response for the *S*.* enterica *strain TA100 only at the higher concentration, with and without S9 mix, and cytotoxic activity for the TA98, TA100, and TA1535, with S9 mix (Table 6). 

### 3.3. Micronucleus Test in the Presence of Megazol Nitro Analogs

Table 7 shows the genotoxicity induction of the nitro analogs in the micronucleus test.

All of the nitro analogs were capable of inducing the formation of micronuclei. However, this induction decreases as the concentration increases (PTAL 05-02 and PAMT 09). Moreover, apoptosis (all analogs) and necrosis (PAMT 09) increase as the concentration increases suggesting there is a cytotoxic activity which inhibits the formation of micronuclei.

## 4. Discussion

The mutagenic and genotoxic assays with the new compounds were designed with recommended *Salmonella enterica *serovar Typhimurium strains and mammalian cell line for an initial toxicology screening [20, 22]. There are several criteria for determining a positive result, such as a concentration-related increase over the range tested, a reproducible increase at one or more concentration in the number of revertant colonies per plate in at least one strain with or without a metabolic activation system, and/or a statistical analysis of a twofold increase over background (induced/spontaneous revertant colonies) response. Moreover, lower nontoxic concentrations are important to be preliminarily determined in order to show the real mutagenic effects of the tested compounds [20, 23].

In this work, the *Salmonella *reverse mutation test with megazol was designed at lower concentrations (0.05 to 1.0 *μ*g/mL) than those reported by other authors [24–26] and taking into account the criteria recommended by OECD [20].

Megazol was able to induce DNA lesions in different sites, since a positive response was found for almost all the tested concentrations and *S. enterica *strains. This is an indicative of base-pair addition (G:C pairs), deletion (G:C pairs), and substitution (G:C for T:A, and A:T for G:C, only with S9 mix). 

Considering the number of highly DNA-reactive products that are formed during megazol metabolism, Farmanguinhos has designed three new synthetic megazol nitro analogs with efficacy against *Trypanosoma cruzi *and lower toxicity [15, 16].

Our results show that the nitro analogs did not present mutagenic activity for most of the *S. enterica *strains (TA97, TA98, TA1535, and TA102). The TA100 was the only responsive strain in the treatment with the nitro analog PTAL 04-09 using a concentration 50 times higher compared to those concentrations used with PTAL 05-02 and PAMT 09.

However, TA100 and TA1535 exhibited cytotoxic activity for most nitro analogs, only in the presence of S9 mix, suggesting that generated metabolites in this condition are more likely to induce base-pair substitutions mutations that lead to cell death. 

Nevertheless, the mutagenic response of the nitro analogs was detected at higher concentration than the lowest megazol concentration to yield mutagenic activity showing the advances made in the development of new substances. 

The structural modifications made on megazol to diminish mutagenic activity and improve antiparasitic potential are reported. The replacement of the megazol's thiadiazole moiety for the triazole moiety on PTAL 05-02 and PAMT 09 and for the pyrazole moiety on PTAL 04-09 is known to improve the trypanocidal activity and reduce nonspecific toxicity [27–30]. Furthermore, the incorporation of a substituent at the C4 position of the imidazole ring, presented on PAMT 09 and PTAL 04-09, might lead to a significant reduction in mutagenic activity, while the trypanocidal activity is moderately retained [31]. Those structural modifications might explain the reduction of mutagenic and cytotoxic activity of the nitro analogs. 

In the genotoxic evaluation (micronucleus test), the analogs were capable of inducing the formation of micronuclei in macrophage cells. In addition, it was important to take into account the mitotic index, since mitosis is a prerequisite for micronucleus formation, and a low mitotic rate might explain the decreased rates of micronucleated cells at the highest concentrations. Furthermore, apoptosis, necrosis, and survival rates suggest cytotoxic activity, which might also be implicated in the low mitotic index and micronucleated cell formation. 

It is reported the cytotoxic activity of megazol by genotoxic assays in cultures without endogenous activation by cytochrome P450 enzymes, while there was not the same activity when present [4]. The use of cytochrome P450 competent cultures might detoxify megazol and the nitro analogs. 

PTAL 04-09 showed no genotoxic activity at the lower concentration and its cytotoxic activity was less prominent compared to other nitro analogs. Its structural modifications might be better suitable for the design of promising new drugs candidate for Chagas' disease treatment. Furthermore, assays with the combination of *Salmonella* metabolically competent and incompetent strains in nitroreductase and acetyltransferases synthesis will be designed in order to elucidate the role of these enzymes in the mutagenicity and cytotoxicity of nitro compounds.

## 5. Conclusion

The demonstration that our compounds exhibited a lower mutagenic, cytotoxic, and genotoxic activity compared to megazol thanks to molecular structure modifications shows that the advances in the rational design of new molecules with lower toxicity and similar pharmacological profile may be of significance in safety evaluations for new drug development.

## Figures and Tables

**Table 1:** Megazol and its nitro analogs condensed formula and IC_50_.

Compound	Condensed formula	IC_50_ (*μ*m)	Reference
Megazol	C_6_H_7_N_6_O_2_S	9.9	[15]
PTAL 05-02	C_6_H_7_N_7_O_2_	552	[15]
PAMT 09	C_7_H_7_F_3_N_7_O_2_	220	[16]
PTAL 04-09	C_7_H_7_N_5_O_2_	120	[16]

**Table 2:** *Salmonella enterica *serovar Typhimurium strains used in this work.

*Salmonella* strain	Reversion event	Genotype	Reference
TA97	Frameshifts	*hisD6610 rfa* Δ(*uvrB chl bio*) pKM101	[18]
TA98	Frameshifts	*hisD3052 rfa *Δ(*uvrB chl bio*) pKM101	[18]
TA100	Base-pair substitutions	*hisG46 rfa* Δ(*uvrB chl bio*) pKM101	[18]
TA1535	Base-pair substitutions	*hisG46 rfa* Δ(*uvrB chl bio*)	[19]
TA102	Transitions/transversions	*hisG428 rfa* pKM101 pAQ1	[18]

**Table 3:** Megazol induction of mutagenicity and cytotoxicity in *Salmonella enterica *serovar Typhimurium standard strains, without (−S9) and with (+S9) metabolic activation.

Strains	Dose (*μ*g/mL)	−S9		+S9	
		Mean ± S.D. (M.I.^a^)	Survival^b^	Mean ± S.D. (M.I.^a^)	Survival^b^
TA97	0	49 ± 19 (1.00)	100	123 ± 26 (1.00)	100
	0.05	90 ± 10 (1.80)	100	176 ± 18 (1.40)	100
	0.1	116 ± 7 (2.30*)	100	168 ± 7 (1.30)	100
	0.2	142 ± 20 (2.80*)	100	273 ± 11 (2.20*)	100
	0.5	165 ± 25 (3.30*)	100	408 ± 7 (3.30*)	80
	1.0	266 ± 31 (5.40*)	100	548 ± 111 (4.40*)	80

TA98	0	27 ± 4 (1.00)	100	40 ± 4 (1.00)	100
	0.05	38 ± 11 (1.30)	92	33 ± 1 (0.80)	100
	0.1	49 ± 5 (1.80)	97	39 ± 7 (0.90)	100
	0.2	81 ± 2 (2.90*)	100	41 ± 14 (1.00)	70
	0.5	211 ± 22 (7.70*)	93	87 ± 2 (2.10*)	75
	1.0	356 ± 35 (13.0*)	**57**	116 ± 14 (2.80*)	77

TA100	0	129 ± 12 (1.00)	100	171 ± 1 (1.00)	100
	0.05	228 ± 13 (1.70)	72	324 ± 47 (1.80)	100
	0.1	366 ± 59 (2.80*)	98	569 ± 58 (3.30*)	100
	0.2	739 ± 29 (5.70*)	70	691 ± 106 (4.00*)	100
	0.5	1302 ± 247 (10.0*)	80	1203 ± 190 (7.00*)	100
	1.0	2109 ± 476 (16.0*)	**57**	2041 ± 319 (11.0*)	**50**

TA102	0	197 ± 88 (1.00)	100	193 ± 77 (1.00)	100
	0.05	280 ± 4 (1.40)	100	408 ± 24 (2.10*)	100
	0.1	289 ± 45 (1.40)	100	459 ± 50 (2.30*)	100
	0.2	185 ± 80 (0.90)	100	594 ± 8.0 (3.00*)	100
	0.5	347 ± 38 (1.70)	100	680 ± 60 (3.50*)	100
	1.0	313 ± 37 (1.70)	100	666 ± 27 (3.40*)	100

^a^Mutagenic index (M.I.): ratio of the *his* ^+^ induced with samples/spontaneous *his* ^+^ in the negative control. S.D.: standard deviation. Positive responses marked in bold when M.I. ≥ 2.

^b^Percent of cellular survival: <70% was considered toxic dose and it is marked in bold.

*Representation of a significant response (*P* ≤ 0.01).

Negative control was DMSO. Positive controls and their M.I., with and without S9 mix, respectively, were 4NQO (10) and 2AA (6.0) for TA97, 4NQO (15) and 2AA (10) for TA98, AS (16) and 2AA (11) for TA100, and MitC (8.7 and 4.3) for TA102.

**Table 4:** PTAL 05-02 induction of mutagenicity and cytotoxicity in *Salmonella enterica *serovar Typhimurium standard strains, without (−S9) and with (+S9) metabolic activation.

Strains	Dose (*μ*g/mL)	−S9		+S9	
		Mean ± S.D. (M.I.^a^)	Survival^b^	Mean ± S.D. (M.I.^a^)	Survival^b^
TA97	0	87 ± 9.8 (1.00)	100	97 ± 25 (1.00)	100
	0.05	89 ± 5.0 (1.03)	90	67 ± 3.5 (0.70)	100
	0.1	89 ± 12 (1.03)	100	68 ± 4.0 (0.70)	92
	0.5	86 ± 8.5 (0.99)	100	74 ± 0.0 (0.76)	90
	1.0	83 ± 1.7 (0.95)	100	69 ± 4.5 (0.71)	80

TA98	0	45 ± 1.5 (1.00)	100	18 ± 6.4 (1.00)	100
	0.05	30 ± 2.1 (0.67)	100	18 ± 5.5 (0.96)	95
	0.1	38 ± 5.0 (0.83)	98	21 ± 2.0 (1.16)	90
	0.5	38 ± 11 (0.84)	100	19 ± 1.7 (1.02)	81
	1.0	58 ± 11 (1.28)	94	22 ± 5.2 (1.18)	91

TA100	0	120 ± 23 (1.00)	100	117 ± 19 (1.00)	100
	0.05	136 ± 15 (1.13)	100	121 ± 25 (1.04)	79
	0.1	102 ± 25 (0.85)	90	106 ± 12 (0.91)	**62**
	0.5	84 ± 10 (0.70)	100	128 ± 18 (1.09)	**64**
	1.0	105 ± 9.8 (0.88)	100	129 ± 13 (1.10)	**49**

TA1535	0	22 ± 3.2 (1.00)	100	5.3 ± 1.5 (1.00)	100
	0.05	21 ± 3.2 (0.94)	100	5.3 ± 0.5 (1.00)	**47**
	0.1	23 ± 2.0 (1.03)	100	5.3 ± 0.5 (1.00)	**42**
	0.5	22 ± 4.0 (1.00)	97	6.6 ± 1.5 (1.25)	**40**
	1.0	26 ± 2.0 (1.15)	93	6.3 ± 2.0 (1.19)	**44**

TA102	0	254 ± 16 (1.00)	100	265 ± 12 (1.00)	100
	0.05	254 ± 31 (1.00)	79	253 ± 19 (0.95)	100
	0.1	276 ± 14 (1.09)	84	225 ± 4.0 (0.85)	100
	0.5	253 ± 11 (1.00)	79	238 ± 16 (0.90)	100
	1.0	261 ± 14 (1.03)	78	256 ± 8.3 (0.97)	100

^a^Mutagenic index (M.I.): ratio of the *his* ^+^ induced with samples/spontaneous *his* ^+^ in the negative control. S.D.: standard deviation. Positive responses marked in bold when M.I. ≥ 2.

^b^Percent of cellular survival: <70% was considered toxic dose and it is marked in bold.

*Representation of a significant response (*P* ≤ 0.01).

Negative control was DMSO. Positive controls and their M.I., with and without S9 mix, respectively, were 4NQO (7.19) and 2AA (9.55) for TA97, 4NQO (10) and 2AA (3.95) for TA98; AS (13) and 2AA (2.10) for TA100, AS (3.31) and 2AA (6.25) for TA1535, and MitC (9.28 and 6.86) for TA102.

**Table 5:** PAMT 09 induction of mutagenicity and cytotoxicity in *Salmonella enterica *serovar Typhimurium standard strains, without (−S9) and with (+S9) metabolic activation.

Strains	Dose (*μ*g/mL)	−S9		+S9	
		Mean ± S.D. (M.I.^a^)	Survival^b^	Mean ± S.D. (M.I.^a^)	Survival^b^
TA97	0	87 ± 9.8 (1.00)	100	97 ± 25 (1.00)	100
	0.05	89 ± 4.5 (1.02)	100	74 ± 5.5 (0.76)	100
	0.1	87 ± 3.6 (1.02)	95	84 ± 7.3 (0.87)	96
	0.5	92 ± 4.9 (1.07)	85	87 ± 2.0 (0.90)	95
	1.0	81 ± 1.4 (0.93)	83	80 ± 8.7 (0.82)	100

TA98	0	45 ± 1.5 (1.00)	100	18 ± 6.4 (1.00)	100
	0.05	58 ± 9.6 (1.28)	100	17 ± 1.0 (0.91)	100
	0.1	66 ± 3.0 (1.46)	93	17 ± 0.5 (0.93)	100
	0.5	50 ± 5.8 (1.10)	97	21 ± 4.0 (1.14)	100
	1.0	48 ± 5.8 (1.07)	78	32 ± 9.0 (1.73)	74

TA100	0	120 ± 23 (1.00)	100	117 ± 19 (1.00)	100
	0.05	100 ± 18 (0.83)	100	104 ± 11 (0.89)	73
	0.1	96 ± 9.5 (0.80)	100	126 ± 12 (1.08)	73
	0.5	95 ± 9.1 (0.79)	100	107 ± 2.6 (0.91)	72
	1.0	100 ± 14 (0.83)	100	107 ± 10 (0.92)	71

TA1535	0	22 ± 3.2 (1.00)	100	5.3 ± 1.5 (1.00)	100
	0.05	19 ± 1.1 (0.94)	100	7.5 ± 0.7 (1.41)	**68**
	0.1	19 ± 2.3 (0.92)	100	9.5 ± 2.1 (1.78)	**55**
	0.5	32 ± 5.5 (1.56)	100	8.6 ± 1.5 (1.63)	**59**
	1.0	25 ± 0.5 (1.21)	85	7.0 ± 1.0 (1.31)	**58**

TA102	0	254 ± 16 (1.00)	100	265 ± 12 (1.00)	100
	0.05	285 ± 30 (1.12)	79	275 ± 17 (1.04)	100
	0.1	289 ± 9.0 (1.14)	76	242 ± 11 (0.91)	100
	0.5	282 ± 28 (1.11)	73	256 ± 10 (0.97)	100
	1.0	307 ± 24 (1.21)	**60**	240 ± 27 (0.91)	100

^a^Mutagenic index (M.I.): ratio of the *his* ^+^ induced with samples/spontaneous *his* ^+^ in the negative control. S.D.: standard deviation. Positive responses marked in bold when M.I. ≥ 2.

^b^Percent of cellular survival: <70% was considered toxic dose and it is marked in bold.

*Representation of a significant response (*P* ≤ 0.01).

Negative control was DMSO. Positive controls and their M.I., with and without S9 mix, respectively, were: 4NQO (7.19) and 2AA (9.55) for TA97, 4NQO (10) and 2AA (3.95) for TA98; AS (13) and 2AA (2.10) for TA100, AS (18) and 2AA (6.25) for TA1535, and MitC (9.28 and 6.86) for TA102.

**Table 6:** PTAL 04-09 induction of mutagenicity and cytotoxicity in *Salmonella enterica *serovar Typhimurium standard strains, without (−S9) and with (+S9) metabolic activation.

Strains	Dose (*μ*g/mL)	−S9		+S9	
		Mean ± S.D. (M.I.^a^)	Survival^b^	Mean ± S.D. (M.I.^a^)	Survival^b^
TA97	0	87 ± 9.8 (1.00)	100	97 ± 25 (1.00)	100
	1.0	85 ± 95 (0.98)	97	74 ± 13 (0.76)	77
	5.0	100 ± 5.7 (1.15)	100	80 ± 13 (0.82)	73
	10	115 ± 16 (1.33)	100	78 ± 16 (0.80)	77
	50	143 ± 11 (1.65)	100	82 ± 9.6 (0.85)	77

TA98	0	45 ± 1.5 (1.00)	100	18 ± 6.4 (1.00)	100
	1.0	66 ± 11 (1.46)	93	18 ± 8.5 (0.98)	100
	5.0	64 ± 3.0 (1.40)	100	17 ± 2.8 (0.91)	100
	10	65 ± 5.1 (1.43)	100	15 ± 1.5 (0.82)	71
	50	66 ± 7.0 (1.45)	100	16 ± 2.0 (0.88)	**62**

TA100	0	120 ± 23 (1.00)	100	117 ± 19 (1.00)	100
	1.0	113 ± 12 (0.94)	100	129 ± 18 (1.11)	**66**
	5.0	165 ± 12 (1.37)	100	144 ± 14 (1.23)	**59**
	10	192 ± 9.4 (1.59)	100	169 ± 9.4 (1.45)	**60**
	50	439 ± 39 (3.64*)	100	297 ± 12 (2.53*)	**55**

TA1535	0	22 ± 3.2 (1.00)	100	5.3 ± 1.5 (1.00)	100
	1.0	21 ± 1.5 (0.94)	97	7.3 ± 1.1 (1.38)	**41**
	5.0	24 ± 2.0 (1.09)	95	8.3 ± 2.3 (1.56)	**41**
	10	25 ± 4.5 (1.10)	100	8.6 ± 1.1 (1.63)	**39**
	50	26 ± 1.5 (1.18)	93	5.6 ± 1.1 (1.06)	**36**

TA102	0	254 ± 16 (1.00)	100	265 ± 12 (1.00)	100
	1.0	258 ± 19 (1.02)	100	266 ± 24 (1.01)	100
	5.0	262 ± 24 (1.03)	100	248 ± 9.1 (0.94)	100
	10	262 ± 31 (1.03)	100	260 ± 30 (0.98)	100
	50	274 ± 16 (1.08)	100	265 ± 7.6 (1.00)	100

^a^Mutagenic index (M.I.): ratio of the *his* ^+^ induced with samples/spontaneous *his* ^+^ in the negative control. S.D.: standard deviation. Positive responses marked in bold when M.I. ≥ 2.

^b^Percent of cellular survival: <70% was considered toxic dose and it is marked in bold.

*Representation of a significant response (*P* ≤ 0.01).

Negative control was DMSO. Positive controls and their M.I., with and without S9 mix, respectively, were: 4NQO (7.19) and 2AA (9.55) for TA97, 4NQO (10) and 2AA (3.95) for TA98; AS (13) and 2AA (2.10) for TA100, AS (18) and 2AA (6.25) for TA1535, and MitC (9.28 and 6.86) for TA102.

**Table 7:** Micronucleus test with RAW 264.7 macrophages using megazol analogs.

Compound	Dose (*µ*g/mL)	‰ Mi.I. ± S.D.	% Apoptosis	% Necrosis	% Survival	% Micronucleus ± S.D.
DMSO		6.0 ± 4.2	0.06	0.12	99.8	0.7 ± 0.4
PTAL 05-02	1.0	0.8 ± 0.0*	5.90*	0.16	93.9*	**3.5 **± 1.2*
	10	3.0 ± 2.2	7.14*	0.02	92.9*	**9.8 **± 6.4*
	100	1.2 ± 0.8*	11.4*	0.06	88.5*	**6.6 **± 2.9*
PAMT 09	1.0	2.8 ± 1.6	2.68	0.00	97.3	**4.0 **±** 4.7**
	10	1.2 ± 0.7*	7.32	0.06	92.6	**2.7 **± 1.7*
	100	1.2 ± 1.7*	24.7*	0.22	75.0*	0.4 ± 0.2
PTAL 04-09	1.0	3.2 ± 2.4	1.50*	0.00	98.5*	1.2 ± 0.8
	10	1.2 ± 1.4*	2.60*	0.00	97.4*	**3.8 **± 1.6*
	100	0.4 ± 1.4*	10*	0.00	90.0*	**3.8 **± 1.9*
MNNG		5.2 ± 3.7	0.30	0.00	99.7*	**2.8 **± 1.4*

Percentage (%) and per mil (‰) mean and standard deviation (S.D.) of the ratio (samples/negative control) values of at least five experiments.

Negative control was DMSO; positive control was MNNG 0.5 *μ*M; Mi.I.: mitotic index; positive responses marked in bold when samples/negative control ratio ≥1.5. *Representation of a significant response (*P* ≤ 0.01).
